# The Association between New-Onset Depressive Symptoms and Participating in Medical Check-Ups among Elderly Individuals

**DOI:** 10.3390/ijerph191811509

**Published:** 2022-09-13

**Authors:** Heejoo Park, Juho Sim, Juyeon Oh, Jongmin Lee, Chorom Lee, Yangwook Kim, Byungyoon Yun, Jin-ha Yoon

**Affiliations:** 1 Department of Business Administration and Data Science, CHA University, 120 Haeryong-ro, Donggyo-dong, Pocheon-si 11160, Korea; 2 Department of Public Health, Graduate School, Yonsei University, Seoul 03722, Korea; 3 Department of Occupational Health, Graduate School of Public Health, Yonsei University, Seoul 03722, Korea; 4 Department of Preventive Medicine, Yonsei University College of Medicine, Seoul 03722, Korea; 5 The Institute for Occupational Health, Yonsei University College of Medicine, Seoul 03722, Korea

**Keywords:** depressive symptoms, medical check-up, elderly, socioeconomic status

## Abstract

The association between adherence to medical check-ups and new-onset depressive symptoms, after adjusting for comprehensive risk factors such as social characteristics, remains unclear. This study aimed to assess the association between mental health and participating in medical check-ups. The survey data of participants aged 60 to 89 were recruited from the seventh Korean Longitudinal Study on Aging. The primary outcome was new-onset depressive symptoms within 2 years measured using the Center for Epidemiologic Studies Depression Scale. Participating in medical check-ups was defined as undergoing biennial medical check-ups. Multivariable logistic regression was performed to estimate adjusted odds ratios (ORs) and 95% confidence intervals (CIs) with consideration of a 2-year time lag. Among 4255 participants, the prevalence of new-onset depressive symptoms was 7.36% (*n* = 313). The prevalence of non-participation in medical check-ups was 11.96% (*n* = 509). The adjusted OR of new-onset depressive symptoms by non-participation in medical check-ups was 1.65 [95% CI 1.22–2.24; *p* = 0.001] after adjusting for various demographic, behavioral, occupational, and social participation characteristics. Our findings demonstrated a significant inverse relationship between participation in medical check-ups and new-onset depressive symptoms. It is necessary to monitor and manage depressive symptoms in vulnerable elderly individuals who do not participate in medical check-ups.

## 1. Introduction

Mental health disorders, especially depression, are among the most serious health concerns. The global prevalence of depression increased by 49.86% between 1990 and 2017 [1]. According to the World Health Organization, depression is a major factor in disability [2]. Depression is a serious mental problem that increases the mortality rate among patients with cardiovascular disease and the risk of fractures [3,4]. Elderly people with depression have an increased risk of suicide [5]. Accordingly, depression is an important mental health problem given its adverse effects on health and quality of life. Moreover, depression has become an important social concern in aging societies.

Compared with young people, elderly individuals have a much higher prevalence of depression [5]. However, elderly individuals with depressive symptoms are reluctant to seek mental health treatment since they believe that the symptoms will naturally improve [6,7,8]. To mitigate the adverse effects of depressive symptoms, there is a need for comprehensive interventions, including primary prevention and diagnosis [9]. Early detection and treatment of depressive symptoms facilitate more effective improvement of depressive symptoms [10]. Periodic medical check-ups might be among the measures that facilitate prompt detection of depression.

In 1980, the national medical check-up program was started in Korea [11]. The Korean National Health Examination Program (National Health Insurance Service) offers free biennial health check-ups to all National Health Insurance subscribers aged > 40 years [12]. The check-ups comprise single blood pressure measurements, blood, and urine tests, chest radiography, and physical measurements including self-administered questionnaires regarding health-related behaviors. A 7-year Taiwanese retrospective cohort study reported that physical examinations facilitated early detection and treatment of target diseases [13].

Moreover, social withdrawal is an important concern in the aging population. In Asia, social withdrawal is an emerging social issue with respect to mental health [14]. Poor social participation can be an early sign of depression. However, the association between adherence to medical check-ups and new-onset depressive symptoms, after adjusting for comprehensive risk factors such as social characteristics, remains unclear.

This study aimed to examine the relationship between mental health and participating in medical check-ups. To explore the bio-social effects of participating in medical check-ups, we adjusted for various demographic, behavioral, occupational, and social participation characteristics. Specifically, we focused on the elderly population using a nationally representative prospective cohort from the Korean Longitudinal Study on Aging (KLoSA). Our findings could inform the prevention of mental health problems in the aging population.

## 2. Methods

### 2.1. Data and Study Design

The KLoSA is a longitudinal survey of Koreans aged ≥45 years living in households selected through multistage stratification probability sampling (regions excluding Jeju Island). The KLoSA uses several questionnaires regarding socioeconomic status, family relationships, health information, and other subjective characteristics. The survey began in 2006 with 10,254 participants and was performed biennially until the latest 8th version in 2020. We initially included 6940 individuals who responded to the 7th survey (2018) and were followed up in the 8th survey. The exclusion criteria were as follows: age <60 or ≥90 years, missing data, baseline depressive symptoms, and no follow-up.

### 2.2. CES-D 10 Depressive Symptoms

The primary outcome was new-onset depressive symptoms within 2 years from the baseline survey. Depressive symptoms were measured using the Center for Epidemiologic Studies Depression Scale (CES-D), which comprises 20 questions; however, we used a 10-item abbreviated scale [15]. The 10 items comprise 8 negative expressions (treated unkindly, sadness, depression, hard, fear, difficulty sleeping, loneliness, feeling heavy with worries) and two positive expressions (get along relatively well, life without dissatisfaction). The CES-D 10 scores were categorized based on the frequency as follows: 0 points (if you feel less than once a day), 1 point (if you thought about it twice a day), 2 points (if you thought about 3–5 times a day), and 3 points (if you thought about it 5–7 times a day). A total score of the 10 CES-D items ≥4 indicates depressive symptoms [16].

### 2.3. Independent Variables and Other Covariates

The independent variable was “participating in medical check-ups”, which was defined as undergoing biennial medical check-ups. All Koreans can get all primary care check-ups biennially free of charge. Furthermore, most paid workers in Korea can participate in medical check-ups with paid sick leave. We adjusted for the following variables: age, sex, education level, smoking, alcohol consumption, economic activity, physical activity, household income, human relationships, residence home ownership, and marital status. Education level was categorized as “middle school or below” and “high school or above”. Smoking was categorized based on the response to the questionnaire item, “Are you currently smoking?” (Yes (smoker) or No (non-smoker)). Alcohol consumption was categorized based on the response to the questionnaire item, “Do you usually drink alcohol?” (Yes (drinker) or No). Economic activity was classified as “Unemployed or not participating” or “Economic activity in progress”.

Physical activity level was classified as “0 times a week,” “≤3 times a week”, “4–5 times a week”, and “≥6 times a week” [17]. Household income, which was defined as the gross household income within the previous year, was separated into three categories: “USD ≤ 19,104”, “USD 19,014–34,236”, and “USD > 34,236”. Human relationships were based on the response to the question “How often do you meet friends or close relationship?”, which was divided into two categories as follows: often (including almost every day, once a week, 2–3 times a week, once a month, and twice a month), and rare (including twice a year, thrice a year, six times a year, almost never within a year, and no one spends time with them). Residence home ownership was categorized as owned and not owned (lump-sum housing lease, monthly rent with deposit, and monthly rent without deposit). Marital status was categorized as “married” and “unmarried” (including bereavement, never married, divorced, separated, or missing).

### 2.4. Statistical Analysis

The frequency and mean (±standard deviation) of the baseline characteristics were calculated according to the presence/absence of depressive symptoms with a 2-year time lag (new-onset depressive symptoms within 2 years). Between-group comparisons of categorical and continuous variables were performed using the chi-square test and *t*-test, respectively. The crude/adjusted odds ratios (ORs) and 95% confidence intervals (CIs) of depressive symptoms were estimated using univariate/multivariable logistic regression models with consideration of a 2-year time lag. Additionally, we investigated between-group differences in the adjusted variables within the adjusted model. Moreover, based on the response to the questionnaire item, “Have you benefited from a free primary medical check-up over the last 2 years?” in the surveys conducted in 2018 and 2020, the participants were stratified into three groups: individuals who participated in biennial medical check-ups (both 2018 and 2020), individuals who participated in a medical check-up in either 2018 or 2020, and individuals who never participated in a medical check-up. We performed a multivariable logistic regression analysis to estimate the adjusted ORs (95% CIs) of new-onset depressive symptoms based on the number of medical check-ups. Furthermore, we performed stratification analyses according to sex, age (young (≤71 years) and old (>71 years)), education, household income, and economic activity to assess the relationship between depressive symptoms and participation in medical check-ups within each stratum. Multivariable logistic regression analysis was used to examine the interaction between medical check-ups and socioeconomic status (income and education levels).

A *p*-value < 0.05 was considered statistically significant for all two-sided statistical tests. All statistical analyses were performed using R (The R Foundation for Statistical Computing, Vienna, Austria, version 4.0.5).

## 3. Results

Among the individuals without depression in the seventh (baseline) survey, 4255 participated in the eighth survey (Figure S1). The prevalence of new-onset depressive symptoms was 7.36% (*n* = 313); further, 88.04% (*n* = 3746) of the individuals participated in medical check-ups. The mean age of the participants was 71.34 ± 7.63 years, with 55.98% (*n* = 2382) of the participants being female. Individuals with new-onset depressive symptoms had a significantly higher prevalence of old age, low education level, non-participation in medical check-ups, unemployment, or non-participation in economic activity, no physical activity, low household income, rare meeting of acquaintances, and lack of home ownership compared with individuals without depressive symptoms (all *p* < 0.001, except 0.004 for education and physical activity, Table 1). Contrastingly, there was no significant between-group difference in sex, smoking status, and alcohol consumption.

The mean (SD) age of participating MC group is 70.9 (7.4), which is younger than the age of the non-participating MC group (*p* < 0.001). The prevalence of more than high school is 40.5% in the participating MC group and 25.9% in the non-participating MC group (*p* < 0.001). The participating MC group show high proportion of alcohol consumption compared to non-participating MC group (2.5% vs. 24.9%, *p* < 0.001). The proportion of economic activity in progress are 65.0% and 73.5% in participation and non-participation MC group, respectively (*p* < 0.001). Proportion of high physical activity per one week (6 times or more), high household income (USD > 34,236), human relationship (often), residence home ownership (owned), and marital status (yes) were higher in participating MC group compare than those of non-participating MC group (12.0% vs. 6.88%, 16.8% vs. 13.8%, 83.1% vs. 78.6%, 87.9% vs. 78.6%, and 77.3% vs. 65.6%, respectively) (Table 2).

According to the logistic regression models, the crude OR (95% CI) of new-onset depressive symptoms with non-participation in medical check-ups was 2.20 [95% CI 1.65–2.93; *p* < 0.001]. Moreover, the adjusted OR (95% CI) of new-onset depressive symptoms with non-participation in medical check-ups in model 3 was ORs 1.65 [95% CI 1.22–2.24, *p* = 0.001] (Table 3).

Furthermore, the adjusted ORs (95% CIs) of depressive symptoms with non-participation and participation in only one medical check-up were 2.32 [95% CI 1.24–3.48, *p* < 0.001] and 1.86 [95% CI 1.39–2.48, *p* < 0.001], respectively, compared with participation in both medical check-ups (Table S1). Interaction analysis revealed a significant association of low income or education in the non-participation in medical check-ups group with new-onset depressive symptoms compared with high income or education in the participation in medical check-ups group (adjusted ORs: 1.81 [95% CI 1.21–2.71; *p* = 0.003] and 1.86 [95% CI 1.23–2.81; *p* = 0.003], respectively, Table 4).

**Figure 1:**
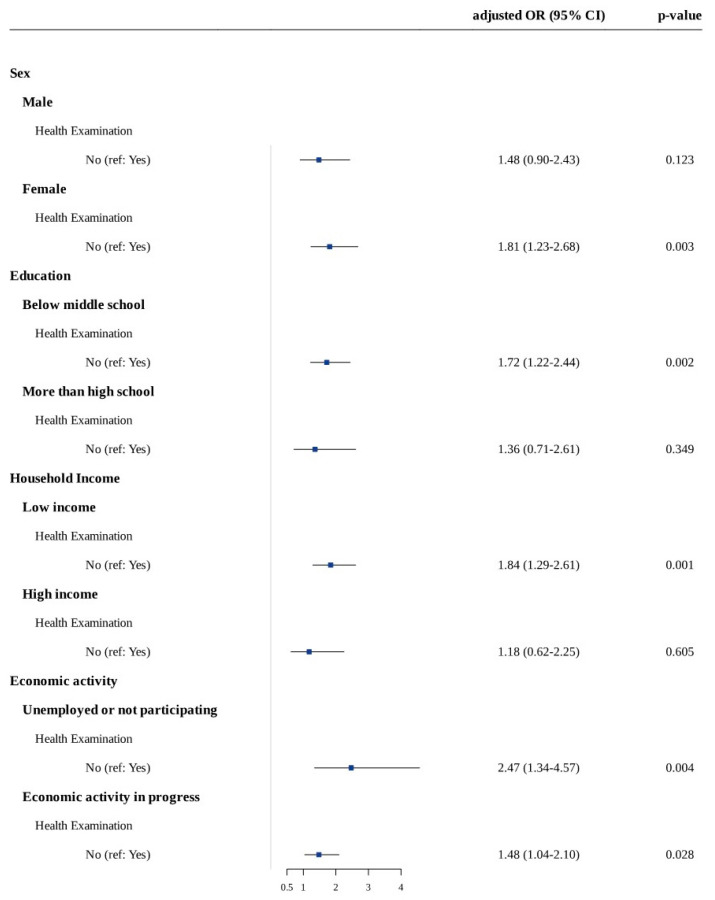
Forest plot of stratification analysis results.

Figure 1 summarizes the results of the stratification analyses. In stratified analysis according to sex, there was a significant relationship between new-onset depressive symptoms and non-participating in medical check-ups in medical check-ups in women (1.81 [95% CI 1.23–2.68; *p* = 0.003]) but not men (1.45 [95% CI 0.90–2.43; *p* = 0.123]). In the stratified analysis according to age, there was a significant relationship between new-onset depressive symptoms and *non-participating* in medical check-ups for both the young and elderly groups (young: adjusted ORs 2.03 [95% CI 1.21–3.38; *p* = 0.007], elderly: adjusted ORs 1.50 [95% CI 1.03–2.18; *p* = 0.036]). In the stratified analysis according to educational level, there was a significant relationship between new-onset depressive symptoms and *non-participating* in medical check-ups at the low educational level (adjusted ORs 1.72 [95% CI 1.22–2.44; *p* = 0.002]) but not at the high education level (adjusted ORs 1.36 [95% CI 0.71–2.61; *p* = 0.349]). In the stratified analysis according to total household income, there was a significant relationship between new-onset depressive symptoms and *non-participating* in medical check-ups in the low household income group (adjusted ORs 1.84 [95% CI 1.29–2.61; *p* = 0.001]) but not at the middle and high household income group (adjusted ORs: 1.75 [95% CI 0.76–4.05; *p* = 0.192] and 0.69 [95% CI 0.25–1.95; *p* = 0.487], respectively). When stratified according to economic activity, non-participation in medical check-ups was significantly associated with new-onset depressive symptoms in both the unemployed and non-participation in economic activities groups (adjusted ORs: 2.47 [95% CI 1.34–4.57; *p* = 0.004]) and the participating in economic activities group (adjusted ORs 1.48 [95% CI 1.04–2.10; *p* = 0.03]).

## 4. Discussion

We found that non-participating in medical check-ups was associated with depressive symptoms in the elderly population. Further, there was a dose-response relationship between depressive symptoms and medical check-ups. Stratification analysis revealed that new-onset depressive symptoms were significantly associated with non-participation in medical check-ups in women, both young and old participants, low education levels, low household income, and participation/non-participation in economic activity. Specifically, there was a stronger relationship between non-participation in medical check-ups and depressive symptoms in the unemployed or not participating in economic activity group as well as in the younger group. Furthermore, our current results highlight that low social and economic status aggravates depressive symptoms in the non-participating MC group. In the current study, low household income or low education with non-participation in MC shows the highest odds of depressive symptoms.

From the perspective of individual health management, consistent medical check-ups could be considered important for mitigating depressive symptoms. This is consistent with previous reports of an inverse relationship between depression and healthy behaviors (preventive health behaviors, self-health management, etc.) [18]. Therefore, it is important to detect and relieve depressive symptoms in the elderly through periodic medical check-ups.

We observed that social interaction had the strongest association with depressive symptoms, which is consistent with previous reports [19,20]. A Japanese prospective cohort study reported that social activities, including interaction with friends and frequency of hobbies, we associated with the odds of depressive symptoms [19]. Moreover, reduced social activity due to medical problems is associated with depressive symptoms [21]. Elderly people can suffer from severe depressive symptoms, especially if they are financially unstable or have a low income level [22]. Additionally, bereaved, separated, or divorced individuals are more likely to experience depressive symptoms [23]. Participants who do not own a house present with more depressive symptoms than homeowners [24]. In the same context, income, house ownership, education level, marital status, economic activity, and human relationships all represent examples of social interactions in our study.

In the elderly population, income and education level are thought to be related to the current socioeconomic status. We found that low income and low education levels were correlated with participating in medical check-ups, which is consistent with previous reports of a correlation between socioeconomic status and participation in medical check-ups [25]. Individuals with higher incomes are more likely to participate in preventative medical check-ups. Additionally, spending time on medical check-ups is considered a burden on elderly individuals who earn income after retirement through part-time, daily work, or self-employment [26]. There have been inconsistent reports regarding the direction of the association between educational level and participation in medical check-ups. In contrast to our findings, Benfeng et al. reported that since highly educated elderly individuals were concerned about their health, they sought high-quality medical check-ups rather than complimentary periodic medical check-ups, which resulted in a low participation rate [27]. Contrastingly, we observed a positive correlation between education level and participation in medical check-ups, which could be attributed to a lack of awareness about health care or ignorance of the presence of medical check-ups.

Moreover, non-participation in medical check-ups within the low socioeconomic status group (low income and education level) was strongly associated with depression since the vulnerable individuals were already exposed to several risk factors regarding their mental health [25]. Complimentary periodic medical check-ups might crucially contribute to maintaining appropriate health status in susceptible individuals through prompt identification and treatment of various diseases. Low socioeconomic status might adversely affect health maintenance in the elderly population, which is consistent with a previous report using nationally representative data [28]. Our interaction analysis results indicate the need for scrutinized investigation of fragile elderly individuals who do not participate in medical check-ups.

Individuals who did not participate in medical check-ups tended to have an unhealthy lifestyle, including smoking, physical inactivity, and lack of social interaction given their marital status and rare meeting of friends, as well as low socioeconomic status. Moreover, these individuals presented with depression symptoms. Elderly individuals with unhealthy lifestyles generally do not seek primary healthcare services [29]. Specifically, physical activity is associated with physical strength, quality of life, and depressive symptoms [20]. Human relationships, including frequency of meeting with friends and marital status, are significantly related to medical check-ups and depressive symptoms [19,30]. This suggests that the absence of someone to care for health maintenance could contribute to non-participation in medical check-ups.

There were differences in participating in MC in wave 7 and wave 8. The non-participating proportion was increased in the eighth wave compared to the seventh wave. It might be a COVID-19 effect, but we have no information about the fear or anxiety about COVID19 infection. So, we cannot conclude whether those increased ratios of non-participating MC and rare human relationships are related to fear of infection from COVID-19 or not. The proportion of rare human relationships is 17.3% in the seventh wave and 16.5% in the eighth wave, and the *p*-value is 0.3883. Hence, there were no changes in rare human relationships between the seventh and eighth waves. Further research with comprehensive information about COVID-19-related psychological status is needed to clarify the relationship.

Aging, aged, and super-aged societies indicate situations where the proportion of individuals aged ≥65 years is >7%, ≥14%, and ≥20% of the total population, respectively [31]. South Korea became an “aging society” in 2000 and an “aged society” in 2018 [32]. Moreover, given the increasing life expectancy among the elderly, it is expected to become a “super-aged society” in 2025 [33]. Furthermore, the percentage of individuals aged ≥65 years is expected to reach 30% and 43.9% in 2036 and 2060, respectively [32,34]. Given the increasingly aging population, several important issues (health, economics, social, etc.) should be carefully considered [35,36]. Our findings demonstrated a meaningful effect of participating in medical check-ups and social activities; accordingly, they may inform prevention measures against mental health disorders.

Our study has the strength of including a relatively large sample from the KLoSA dataset, which is representative of the health-related variables and sociodemographic factors of the Korean population. To our knowledge, this is the first Asian nationwide study to examine the relationship between depressive symptoms and participation in medical check-ups in the elderly population, which elucidated the temporal relationship with a 2-year time lag. However, this study has several limitations. First, this was a cross-sectional study; therefore, we could not determine the causal relationships between depressive symptoms and participation in medical check-ups. Second, we did not consider various factors (including thyroid function, insomnia, and other mental diseases) that can influence depression, which may have acted as unmeasured confounders. Third, survey research is susceptible to memory and recall bias. Fourth, the household income should be adjusted by the number of economically active as well as dependent family members. However, adjusted household incomes were not included in the current study because of a lack of information about family members’ economic statuses.

## 5. Conclusions

Our findings demonstrated a significant inverse relationship between participation in periodic medical check-ups and new-onset depressive symptoms. Specifically, new-onset depressive symptoms were significantly associated with non-participation in medical check-ups in women; both young and old individuals; and individuals with low education levels, low household income, and low economic activity. It is necessary to monitor and manage depressive symptoms in vulnerable elderly individuals who do not participate in medical check-ups.

## Figures and Tables

**Table 1:** Baseline characteristics of participants by new-onset of depressive symptoms.

Variable	Depressive Symptom (*n* = 313)	Non-Depressive Symptom (*n* = 3942)	*p*-Value
Age			<0.001
Mean (SD)	73.45 (7.9)	71.17 (7.6)	
Sex			0.327
Men	129 (41.2%)	1744 (37.5%)	
Women	184 (58.8%)	2198 (62.5%)	
Education level			0.004
Middle school or below	213 (68.1%)	2393 (60.7%)	
High school or above	100 (32.0%)	1549 (39.3%)	
Smoking			0.104
Yes	20 (6.4%)	367 (9.3%)	
No	293 (93.6%)	3575 (90.7%)	
Alcohol consumption			0.116
Yes	86 (27.5%)	1259 (31.9%)	
No	227 (72.5%)	2683 (68.1%)	
Participating medical check-up			<0.001
Yes	245 (78.3%)	3501 (88.1%)	
No	68 (21.7%)	441 (11.9%)	
Economic activity			<0.001
Economic activity in progress	79 (25.2%)	1366 (34.7%)	
Unemployed or not participating	234 (74.8%)	2576 (65.4%)	
Physical activity (1 week)			0.004
0	225 (71.9%)	2503 (63.5%)	
≤3	21 (6.7%)	482 (12.2%)	
4–5	30 (9.6%)	508 (12.9%)	
≥6	37 (11.8%)	449 (11.4%)	
Household Income *			<0.001
USD ≤ 19,014	159 (50.8%)	1543 (39.1%)	
USD 19,014–34,236	76 (25.2%)	1197 (30.4%)	
USD > 34,236	78 (24.9%)	1202 (30.5%)	
Human relationship			<0.001
Often	208 (66.5%)	3376 (85.6%)	
Rare	105 (33.6%)	566 (14.4%)	
Residence home ownership			<0.001
Owned	239 (76.4%)	3455 (87.7%)	
Not Owned	74 (23.7%)	487 (12.4%)	
Marital status			<0.001
Yes	210 (67.1%)	3020 (76.6%)	
No	103 (32.9%)	922 (23.4%)	

Residence home ownership, no: long-term rent, monthly rent, etc.; marital status, no: separation, divorce, bereavement, disappearance, unmarried. *: USD 1 = KRW 1314. Values are expressed by *n* (%) or mean (SD).

**Table 2:** Baseline characteristics of participants by medical check-up (MC).

Variable	Participating MC (*n* = 3746)	Non-Participating MC (*n* = 509)	*p*-Value
Age			<0.001
Mean (SD)	70.90 (7.5)	74.56 (8.2)	
Sex			0.139
Men	1665 (44.5%)	208 (40.9%)	
Women	2081 (55.6%)	301 (59.1%)	
Education level			<0.001
Middle school or below	2229 (59.5%)	377 (74.1%)	
High school or above	1517 (40.5%)	132 (25.9%)	
Smoking			0.178
Yes	332 (8.9%)	55 (10.8%)	
No	3414 (91.1%)	454 (89.2%)	
Alcohol consumption			<0.001
Yes	1218 (32.5%)	127 (25.0%)	
No	2528 (67.5%)	382 (75.1%)	
Economic activity			<0.001
Economic activity in progress	2436 (65.0%)	374 (73.5%)	
Unemployed or not participating	1310 (35.0%)	135 (26.5%)	
Physical activity (1 week)			<0.001
0	2342 (62.5%)	386 (75.8%)	
≤3	459 (12.3%)	44 (8.6%)	
4–5	494 (13.2%)	44 (8.6%)	
≥6	451 (12.0%)	35 (6.9%)	
Household Income *			<0.001
USD ≤ 19,014	2213 (59.1%)	352 (69.2%)	
USD 19,014–34,236	905 (24.2%)	87 (17.1%)	
USD > 34,236	628 (16.8%)	70 (13.8%)	
Human relationship			<0.001
Often	3112 (83.1%)	472 (78.6%)	
Rare	551 (16.9%)	120 (21.4%)	
Residence home ownership			<0.001
Owned	3294 (87.9%)	400 (78.6%)	
Not Owned	452 (12.1%)	109 (21.4%)	
Marital status			<0.001
Yes	2896 (77.3%)	334 (65.6%)	
No	850 (22.7%)	175 (34.4%)	

Residence home ownership, no: long-term rent, monthly rent, etc.; marital status, no: separation, divorce, bereavement, disappearance, unmarried. *: USD 1 = KRW 1314.

**Table 3:** The ORs (95% CIs) of new-onset of depressive symptoms by participating in medical check-ups among the elderly population in the logistic regression model.

Variable	Values	Crude Model			Model 1			Model 2			Model 3		
		Odds Ratios	CI	*p*	Odds Ratios	CI	*p*	Odds Ratios	CI	*p*	Odds Ratios	CI	*p*
Participating medical check-up	Yes	1.00 (reference)			1.00 (reference)			1.00 (reference)			1.00 (reference)		
	No	2.2	1.65–2.93	<0.001	1.95	1.46–2.61	<0.001	1.84	1.34–2.53	0.001	1.65	1.22–2.24	0.001

Model 1: adjusted by age and sex. Model 2: model 1 + adjusted by residence home ownership, education, marital status, economic activity, human relationship, smoking, alcohol consumption, and physical activity. Model 3: model 2 + adjusted by household income level.

**Table 4:** Participating in medical check-ups over the past 2 years according to SES.

Medical Check-Up	Socioeconomic Status	Final Model ^¶^		
		Odds Ratios	CI	*p*
Yes	High household income	1.00 (reference)		
	Low household income	0.95	0.71–1.28	0.75
No	High household income	1.21	0.65–2.25	0.55
	Low household income	1.78	1.18–2.67	0.006
Yes	High education	1.00 (reference)		
	Low education	1.09	0.80–1.49	0.57
No	High education	1.43	0.76–2.69	0.27
	Low education	1.89	1.25–2.87	0.002

^¶^ Adjusted by sex, age, physical activity, marital, smoking, alcohol consumption, residence home ownership, economic activity, and human relationship. Abbreviation: CI; confidence interval.

## Data Availability

Data are available in a publicly accessible repository that does not issue DOIs (https://survey.keis.or.kr/klosa/klosadownload/List.jsp accessed on 10 March 2022).

## Supplementary Materials

The following are available online at: https://www.mdpi.com/article/10.3390/ijerph191811509/s1, Figure S1: Flow chart of participants selection process, Table S1: The dose-dependent association between new-onset depressive symptoms and participation in medical check-ups.
