# Supplementary material for: The Association between New-Onset Depressive Symptoms and Participating in Medical Check-Ups among Elderly Individuals
Source: Int J Environ Res Public Health. 2022 Sep 13;19(18):11509. doi: 10.3390/ijerph191811509 (PMC9516985; doi:10.3390/ijerph191811509)
Supplement: Supplementary file 1 [file ijerph-19-11509-s001.zip › ijerph-1893200-supplementary.pdf]

Table S1. The dose-dependent association between new-onset depressive symptoms and participation in medical check-ups.

Variable	Values	Crude Model			Final Model ¶		
		Odds Ratios	CI	*p*	Odds Ratios	CI	*p*
Number of Participating medical check-up in 2 years	Twice	1.00 (reference)			1.00 (reference)		
	Once	2.14	1.62–2.82	<0.001	1.86	1.39–2.48	<0.001
	None	3.57	2.45–5.19	<0.001	2.32	1.24–3.48	<0.001

¶ Adjusted by Sex, Age, Physical activity, Education level, Marital, Smoking, Alcohol consumption, Residence home ownership, Household income, Economic activity and Human relationship. Abbreviation: CI; confidence interval.

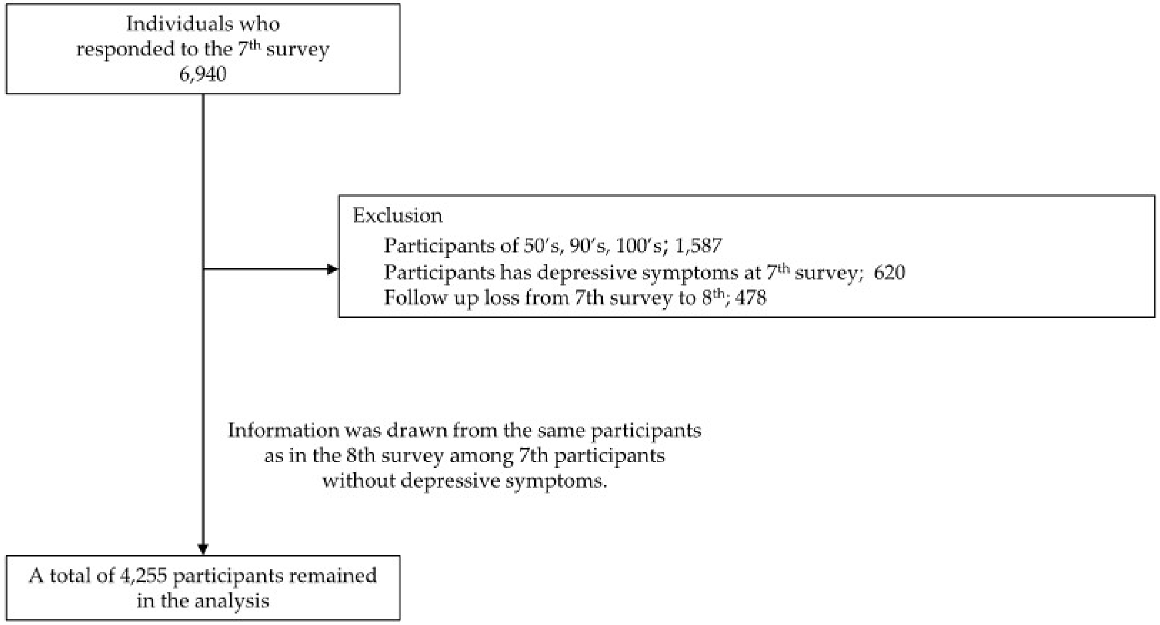

Figure S1. Flow chart of participants selection process.
